# Outcomes following surgical management of patellar instability in hypermobile patients are favourable compared to non‐operative management in non‐hypermobile patients: A systematic review and meta‐analysis

**DOI:** 10.1002/jeo2.70256

**Published:** 2025-06-01

**Authors:** Joshua Dworsky‐Fried, Benjamin Blackman, Dan Cohen, Devin Peterson, Olufemi R. Ayeni, Volker Musahl, Darren de SA

**Affiliations:** ^1^ Michael G. deGroote School of Medicine McMaster University Hamilton Ontario Canada; ^2^ School of Medicine University of Limerick Limerick Ireland; ^3^ Division of Orthopaedic Surgery, Department of Surgery McMaster University Hamilton Ontario Canada; ^4^ Department of Orthopaedic Surgery, UPMC Freddie Fu Sports Medicine Center University of Pittsburgh Pittsburgh Pennsylvania USA

**Keywords:** Beighton score, dislocation, MPFL reconstruction, hypermobility, patella, surgical management

## Abstract

**Purpose:**

To assess the outcomes of surgical management of patellar instability in hypermobile patients.

**Methods:**

Three online databases (PubMed, MEDLINE and EMBASE) were searched from inception to 27 September 2024, to identify studies investigating the surgical management options for patellar instability in hypermobile patients. Data pertaining to patient demographics, patient management, redislocation rates and Kujala scores were abstracted. Weighted means and meta‐analyses were conducted to compare rates of redislocation as well as post‐operative Kujala scores. However, data pooling was not performed in cases of high heterogeneity. The quality of included studies was assessed using the MINORS criteria.

**Results:**

A total of nine studies and 303 patients were included in this review. The pooled mean post‐operative redislocation rate was 9% at a mean follow‐up time of 45.4 months. The mean post‐operative redislocation rate ranged from 7.3% to 28.5% following medial patellofemoral reconstruction (MPFLR). The mean post‐operative Kujala score ranged from 64.3 to 95.3. The post‐operative complication rate was 11.7%.

**Conclusion:**

This systematic review demonstrated that surgical management, particularly MPFLR, of patellar instability in hypermobile patients may result in lower redislocation rates and favourable post‐operative outcomes compared to non‐operative management in non‐hypermobile patients. The current available literature for this patient population is highly heterogeneous, indicating the need for high‐quality studies to more accurately assess intrinsic risk factors and surgical techniques.

**Level of Evidence:**

Level IV.

**Abbreviations**
AKPS: Anterior Knee Pain Scale
CI: confidence interval
EDS: Ehlers–Danlos syndrome
GJH: generalized joint hypermobility
JHS: joint hypermobility syndrome
MINORS: methodological index for non‐randomized studies
MPFLR: medial patellofemoral ligament reconstruction
PROM: patient‐reported outcome measure
SD: standard deviation
TTO: tibial tubercle osteotomy
TT‐TG: tibial tuberosity to trochlear groove
VMO: vastus medialis oblique

## INTRODUCTION

Patellar dislocations comprise nearly 3% of knee injuries [10, 19], with the majority occurring in adolescents [10, 19, 52]. Various intrinsic bone and soft‐tissue related risk factors predispose individuals to patellar dislocations, such as trochlear dysplasia, increased tibial tubercle to trochlear groove (TT‐TG) distance, patellar tilt, and femoral anteversion [7, 51]. Patients with hypermobility, idiopathic or associated with an underlying connective tissue disorder such as Ehlers–Danlos Syndrome (EDS), have an increased risk of patellar dislocations and instability [4, 34, 52, 56]. The Beighton Score is a standardized score consisting of five manoeuvres, with a score of ≥5 out of 9 in adults or ≥6 out of 9 in children indicating a diagnosis of hypermobility [31, 47]. Hypermobility results in increased ligamentous laxity, allowing joint movement beyond normal limits [55]. In these cases, the medial constraints of the knee, most notably the medial patellofemoral ligament (MPFL), are less resistant to lateral translation of the patella. The increased laxity makes the MPFL more susceptible to failure, leading to recurrent patellar dislocations.

Generalized joint hypermobility (GJH) and joint hypermobility syndrome (JHS), such as EDS and Down Syndrome, are known to be predisposing factors to a range of musculoskeletal disorders, including patellofemoral instability. Consequently, patients with hypermobility experience an increased incidence of isolated and recurrent patellar dislocation and knee pain [14, 18, 25, 36, 49]. Patients with hypermobility often experience recurrent patellar dislocations before reaching skeletal maturity, putting them at increased risk for long‐term pain, arthritis and loss of function/sport participation [23, 53]. This presents a unique challenge for surgeons, as it is unclear how effective conventional procedures are in treating instability in patients with hypermobility. Recent systematic reviews have highlighted the success of early surgery following patellar dislocation to prevent the risk of long‐term instability [3, 6, 28], which offers particular relevance to patients with intrinsic risk factors such as hypermobility. Surgery for patellar dislocation often involves addressing the MPFL through repair [17] or reconstruction [34]. These may be combined with additional procedures such as lateral retinacular release [32, 33], trochleoplasty [8, 44], vastus medialis oblique (VMO) advancement [21], medial imbrication [45] and tibial tubercle osteotomy (TTO) [11], depending on the patient's anatomy and intrinsic risk factors.

In the current literature, there is scarce and conflicting evidence assessing the functional outcomes following surgical management of patellar instability in hypermobile patients [14, 15, 18, 25]. Given their intrinsic risk factors, including increased ligamentous laxity and a higher predisposition to recurrent instability, this review aims to assess the management of patellar instability in patients with hypermobility to determine whether they experience different results compared to non‐hypermobile individuals. We hypothesize that MPFL reconstruction (MPFLR) will be the most used and effective technique, and allografts will result in lower dislocation rates. MPFLR has been shown to be effective in managing patellar dislocations in paediatrics and adults [3, 6]; therefore, we hypothesize that MPFLR will be the most used and effective technique in this review. Due to potential collagen defects in hypermobile patients, we hypothesize that allografts will be the most effective graft choice and autografts will have a higher failure rate.

## MATERIALS AND METHODS

The Preferred Reporting Items for Systematic Reviews and Meta‐analyses guidelines for coordinating and reporting systematic reviews were followed during the development of this research [29].

### Search criteria

Three online databases (PubMed, MEDLINE and EMBASE) were searched from inception to 29 September 2024, to identify studies investigating the management of patellar instability in patients with hypermobility. Comprehensive search terms were used, including ‘MPFL’, ‘medial patellofemoral ligament’, ‘patellar instability’, ‘patellar dislocation’, ‘repair’, ‘reconstruction’, ‘treatment’, ‘connective tissue disorder’, ‘hyper flexibility’, ‘joint instability’, ‘hypermobile’, ‘hypermobility’, ‘laxity’, and ‘Ehlers‐Danlos syndrome’ (Supporting Information S1: Table 1).

The research question and study eligibility were determined a priori. Studies were selected for inclusion if they met the following criteria: (1) treatment of patellar dislocations/instability, (2) patient with documented hypermobility [31] (Beighton score ≥5 in adults or ≥6 in children) or connective tissue disorder, (3) level of evidence I–IV, (4) clinical and/or functional outcomes reported, (4) human studies and (5) studies published in the English language. Exclusion criteria included (1) patients without hypermobility (Beighton <4), (2) textbook chapters, (3) conference abstracts, (4) biomechanical or cadaveric/animal studies and (5) case studies and case series with five or fewer patients. The reference lists of included studies and pertinent review papers were manually searched to ensure all means of study identification were exhausted.

### Screening

Two authors independently screened titles and abstracts, and conflicts were resolved through consensus or consultation with a more senior author if no consensus was reached. During the full‐text screening stage, studies were independently screened by the initial two authors, and disagreements were resolved in the same manner. Screening was performed using the Cochrane software.

### Assessment of agreement

Inter‐reviewer agreement was evaluated using the *κ*‐statistic for screening. Agreement was defined as follows: *κ* of 0.91–0.99 was considered to be almost perfect agreement; *κ* of 0.71–0.90 was considered to be considerable agreement; *κ* of 0.61–0.70 was considered to be high agreement; *κ* of 0.41–0.60 was considered to be moderate agreement; *κ* of 0.21–0.40 was considered to be fair agreement; and a *κ* value of 0.20 or less was considered to be no agreement [26].

### Quality assessment

The methodological quality of non‐randomized studies was evaluated using the methodological index for non‐randomized studies (MINORS) criteria [46]. The MINORS criteria were chosen due to their validation and widespread use in evaluating non‐randomized surgical studies. Using the items on the MINORS checklist, non‐comparative studies can achieve a maximum score of 16, while comparative studies can achieve a maximum score of 24. Non‐comparative studies were categorized a priori as follows: 0–4 indicates very low‐quality evidence, 5–7 indicates low quality, 8–12 indicates fair quality and scores ≥13 indicate high quality. For comparative studies, categorization was determined a priori as follows: 0–6 very low quality, 7–10 low quality, 11–15 fair quality, 16–20 good quality and ≥20 high quality [6]. Individual MINORS scores can be seen in Table 1.

**Table 1:** Study characteristics and outcomes.

Author (year)	Level of evidence	Mean MINORS score	Number of patients/knees	Primary surgical procedure	Graft (s) used (*n*)	Mean follow‐up time (months)	Lost to follow‐up (%)	Female (%)	Mean age (years)	Mean Beighton Score	Diagnosis of joint hypermobility syndrome (JHS) (%)	Redislocation rate (%)	Mean Kujala score	Reported statistical associations
Abouelsoud (2015)	IV		16/16	Anatomic physeal‐sparing MPFLR	QT autograft: 16	29.3	0	68.8	11.5	7	NR	0	Preoperative: 56 Post‐operative: 94 *p* < 0.005	NR
Hiemstra (2021)	III		92/92	MPFLR	HT autograft: 92	24.5	8	78.3	22.9	NR (all >4)	NR	3.3	NR	Beighton score and post‐operative outcomes: *p* = NS Beighton score and functional testing: *p* = NS Beighton score and age: *p* = 0.05 Beighton score and female sex: *p* = 0.01 Beighton score and presence of knee hyperextension: *p* < 0.001
Howells (2012)	III		25/25	MPFLR	Semitendinosus autograft: 25	15.0	0	92	25.4	NR (all >6)	JHS: 32	0	Preoperative: 46.6 Post‐operative: 64.3 *p* = 0.018	NR
Imerci (2022)	II		11/17	MPFLR, TTO (Fulkerson method)	Semitendinosus allograft: 17	26.4	0	63.6	14.8	NR	EDS: 36.36 Down syndrome: 18.2 trichorhinophalangeal syndrome: 9.1 McCune Albright syndrome: 9.1% Klippel–Feil syndrome: 9.1% Generalized joint hypermobility: 18.2	0	Preoperative: 56 Post‐operative: 86 *p* < 0.001	NR
Joo (2007)	IV		5/6	LR, proximal realignment of patella, semitendinosus tenodesis, transfer of patellar tendon	NR	54.5	0	100	6.1	NR	Down syndrome: 40 William's syndrome: 20	0	Preoperative: NR Post‐operative: 95.3	NR
Niedzielski (2015)	IV		11/11	VMO advancement, LR, a Roux‐Goldthwait partial patellar ligament transposition and Galeazzi semitendinosus tenodesis	NR	97.2	0	63.4	13.8	6.2	NR	9.1	NR	NR
Parikh (2024)	IV		31/47	MPFLR	HT allograft: 16 Gracilis autograft: 17 Semitendinosus autograft: 13 QT autograft: 1	86.4	0	87.1	14.9	6.8	EDS: 100	19.1 Autograft: 22.6 Allograft: 12.5	Preoperative: NR Post‐operative: 75 Autograft: 74.1 Allograft: 76.2	No significant difference between PROMs and Beighton score (<7 or >7): *p* = NS
Reddy (2022)	IV		57/76	MPFLR	Allograft (unspecified): 76	36.0	0	75	14	7	EDS: 3.5 Down syndrome: 5.3 Cerebral palsy: 3.5 Jacobsen syndrome: 1.8 Severe learning disability: 3.5	9	Preoperative: NR Post‐operative: 89	No difference in complications in syndromic vs. non‐syndromic patients: *p* = NS
Trisolino (2023)	II		55/74	Modified Roux‐Goldthwait, VMO advancement, LR	NR	39.6	8.1	64	10.2	NR	EDS: 7.3 Down syndrome: 25.5 Multiple epiphyseal dysplasia/spondyloepiphyseal dysplasia: 7.3 DiGeorge syndrome: 3.6 Jacobsen syndrome: 1.8 Prune belly syndrome: 1.8 Marfan syndrome: 1.8 Type I osteogenesis imperfecta: 1.8 Prader–Willi syndrome: 1.8 Coffin–Siris syndrome: 1.8 Cerebral palsy: 12.7 Muscular dystrophy: 1.8 Amyoplasia congenital: 9.1 Torsional malalignment: 3.6 Sequelae of tibial and femoral bone infection during infancy: 3.6 Sotos syndrome: 1.8 Moebius syndrome: 1.8 VACTERL syndrome: 1.8 Nail‐patella syndrome: 1.8 Congenital pseudarthrosis of tibia in NF1: 1.8 Tibial hemimelia: 5.5	19.1	Preoperative: NR Post‐operative: 81.4	Congenital ligamentous laxity and increased risk of dislocation: *p* = 0.001 Congenital ligamentous laxity and decreased 10‐year survival rate (free from redislocation): *p* = 0.018

Abbreviations: EDS, Ehlers–Danlos syndrome; HT, hamstring tendon; LR, lateral release; NF1, neurofibromatosis type I; NR, no result; NS, non‐significant; PROM, patient‐reported outcome measure; QT, quadriceps tendon; VMO, vastus medialis oblique.

### Data abstraction

Data were extracted in an electronic spreadsheet designed a priori (Google Sheets; Google LLC). Extracted data included study characteristics (e.g., author(s), year of publication and level of evidence) and demographic data (e.g., number of patients, patient age and sex), follow‐up time, and number lost to follow‐up. Anatomic details were also recorded (e.g., presence of loose bodies, trochlear dysplasia, Beighton score, diagnosis of hypermobility, and Insall‐Salvati score). The surgical technique (e.g., MPFLR, lateral retinacular release and Roux–Goldthwait), graft type (e.g., semitendinosus and quadriceps) and outcomes (i.e., redislocation rate, Kujala score and complications) were also included in the extraction.

### Outcome reporting and statistics

The primary outcomes were the redislocation rates and Kujala scores. Secondary outcomes included other patient‐reported outcome measures (PROMs) and complications. Single‐arm meta‐analyses with MPFLR, non‐MPFLR, allograft and autograft subgroups were conducted to assess the pooled redislocation rates and mean post‐operative Kujala scores. Given that results from included studies were not stratified by other demographic features (e.g., skeletal maturity), and data such as preoperative Kujala scores were not reported, additional subgroup analysis was not performed. The meta‐analyses were performed using a random effects model (RStudio Version 2024.09.1+394). The Kujala Anterior Knee Pain Scale (AKPS) is a 13‐item patient‐reported assessment designed to assess patellofemoral pain in adolescents and young adults. The Kujala questionnaire is scored out of 100 points, with a lower score indicative of more subjective symptoms and functional limitations [24]. Forest plots were created to pool studies across the same outcome measures (RStudio Version 2024.09.1+394). The *I* ^2^ test was used to assess heterogeneity. Values of *I* ^2^ between 25%–49% and 50%–75% were considered ‘moderate’ and ‘high’ statistical heterogeneity, respectively [54]. If the heterogeneity was considered ‘high’, data pooling was avoided. Aside from redislocation rate, complications were classified using the Clavien–Dindo classification system [5].

## RESULTS

### Literature search

**Figure 1:**
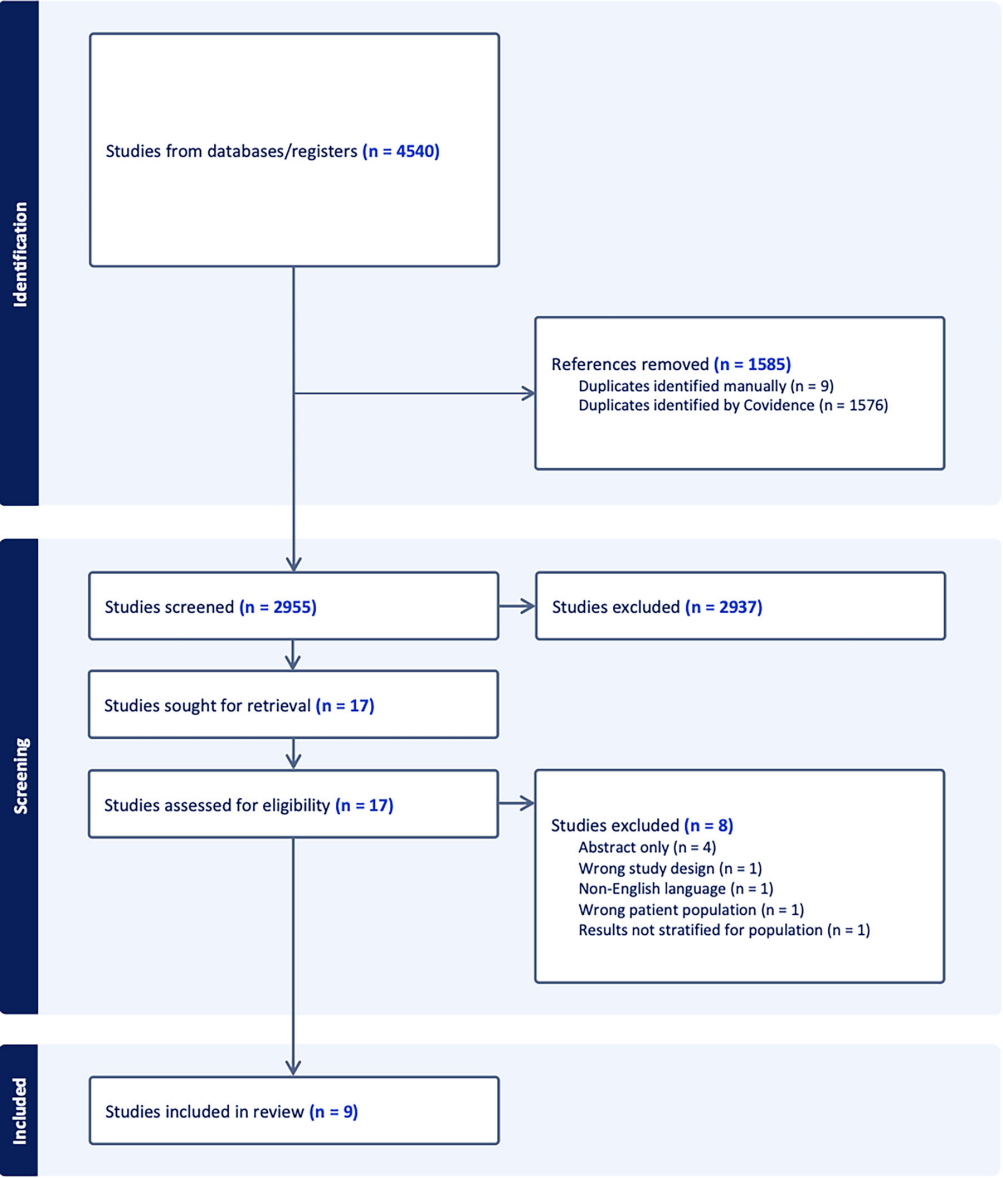
Preferred Reporting Items for Systematic Reviews and Meta‐analyses flow diagram representing a systematic review on management of patellar instability in hypermobile patients.

The initial literature search yielded 4540 studies, of which 1585 duplicates were removed. Among the remaining 2955 unique articles, 2937 were removed following title and abstract screening. Systematic screening and assessment of eligibility yielded nine full‐text studies that satisfied inclusion criteria (Figure 1). Inter‐rater reliability analysis showed that considerable agreement was achieved at both the title and abstract screening stage (*κ* = 0.74, 95% confidence interval [CI]: 0.55–0.93) and full‐text (*κ* = 0.88, 95% CI: 0.66–1.0) stages of screening.

### Study quality

Among the nine studies included in this review, five (55.6%) were Level IV evidence [1, 22, 35, 40, 41], two (18.2%) were Level III evidence [15, 18] and two (18.2%) were Level II evidence [20, 50]. Across eight non‐comparative studies, the mean MINORS scores were determined to be 11.5 (SD: 0.9, range: 10–13) [1, 15, 20, 22, 35, 40, 41, 55]. A score of 19 was assigned to the one included comparative study [18].

### Study characteristics

This review evaluated nine studies, which included 303 patients with hypermobility and/or a positive Beighton score who underwent surgical management for either acute or recurrent patellar instability (Table 1) [1, 15, 18, 20, 22, 35, 40, 41, 50]. Included patients had a weighted mean age of 16.8 (SD: 6.0) years, reported in nine studies [1, 15, 18, 20, 22, 35, 40, 41, 50]. The overall weighted proportion of female patients was 75.9% (SD: 13.5) [1, 15, 18, 20, 22, 35, 40, 41, 50]. The weighted mean follow‐up time was 45.4 (SD: 30.4, range: 15−97.2) months, and the weighted mean percentage of patients lost to follow‐up was 3.9% (SD: 3.5) [1, 15, 18, 20, 22, 35, 40, 41, 50]. The overall weighted proportion of skeletally immature patients was 73.07 (SD: 46.2)%, reported in six studies [1, 20, 22, 35, 40, 50]. When reported, 164 (85.9%) patients were reported to have recurrent patellar dislocations [1, 15, 18, 40], whereas 27 (14.1%) patients experienced first‐time dislocations [20, 22, 35]. The mean weighted preoperative Beighton score was 6.9 (SD: 0.4, range: 6.2−7), as reported in four studies. Two studies reported that all included patients had a Beighton score ≥4 [15] or ≥7 [18], respectively. A wide range of concurrent syndromes were reported across the included studies. Full details regarding study characteristics and patient demographics can be found in Table 1.

### Surgical procedures

Across the nine studies, various operations and conservative management protocols are described. Surgical management involved MPFLR [1, 15, 18, 20, 40, 41], Elmslie‐Trillat procedure [41], Fulkerson procedure [20], trochleoplasty [15, 41], lateral release [20, 22, 35, 50], meniscectomy [20], Roux–Goldthwait procedure [35, 50] and semitendinosus tenodesis [22, 35]. Two studies reported on conservative management plans, which consisted of bracing and strengthening exercises [15, 22]. Among included studies that reported data, 109 knees (39.9%) were managed with surgery utilizing allograft [20, 40, 41], whereas autografts were used in 164 knees (60.1%) [1, 15, 18, 40]. Post‐operative rehabilitation protocols varied widely across studies. Complete surgical and rehabilitation details are found in Table 1.

### Post‐operative redislocation rates

**Figure 2:**
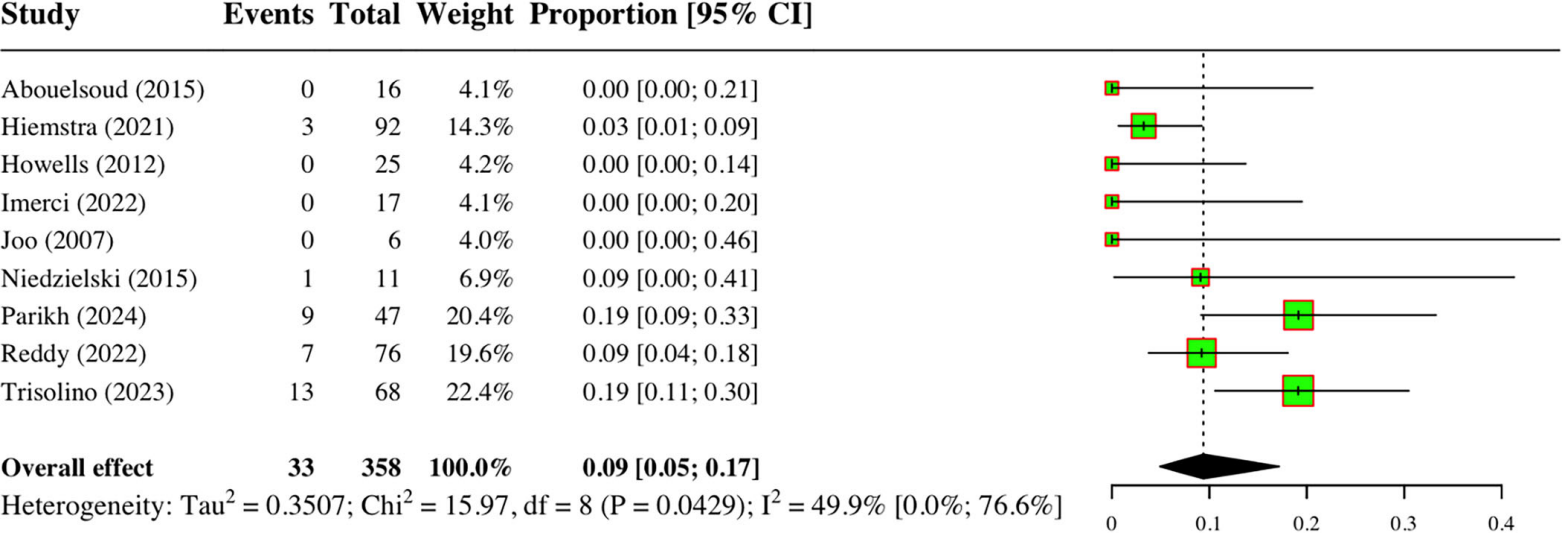
Forest plot (random effects) showing the overall pooled rates of redislocation and heterogeneity of outcomes across included patients with hypermobility.

**Figure 3:**
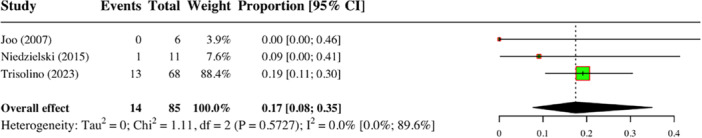
Forest plot (random effects) showing the overall pooled rates of redislocation and heterogeneity of outcomes across included patients with hypermobility managed with non‐MPFLR procedures. MPFLR, medial patellofemoral ligament reconstruction.

**Figure 4:**
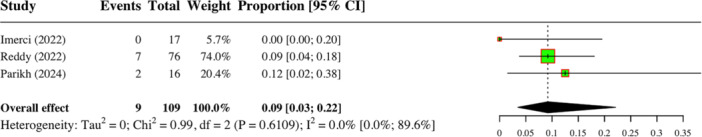
Forest plot (random effects) showing the overall pooled rates of redislocation and heterogeneity of outcomes across included patients with hypermobility managed with allografts.

The primary outcome reported in the included studies was the redislocation rate. A single‐armed meta‐analysis was conducted on nine studies comprising 303 patients (364 knees) which showed a pooled mean post‐operative redislocation rate of 9% (*p* = 0.04, 95% CI: 5%–17%, *I* ^2^ = 49.9%) [1, 15, 18, 20, 22, 35, 40, 41, 50] (Figure 2). When stratified by surgical procedure, the mean post‐operative redislocation rate from six studies comprising 232 patients (273 knees) who underwent MPFLR ranged from 7.3% to 28.5%; however, data pooling was not performed due to high heterogeneity [1, 15, 18, 20, 40, 41]. The mean post‐operative redislocation rate among patients who underwent non‐MPFLR procedures was 17% (*p* = 0.6, 95% CI: 8%–35%, *I* ^2^ = 0%), reported in three studies comprising 71 patients (85 knees) (Figure 3) [22, 35, 50]. A meta‐analysis assessing allografts was conducted on three studies comprising 109 knees, which showed a pooled mean redislocation rate of 9% (*p* = 0.6, 95% CI: 3%–22%, *I* ^2^ = 0%) (Figure 4) [20, 40, 41]. The overall mean redislocation rate among patients who underwent surgical management with autografts ranged from 2% to 23%, reported in five studies comprising 175 knees; data pooling was not performed due to high heterogeneity [1, 15, 18, 35, 40].

### Kujala score

Data pooling for post‐operative Kujala scores was not conducted due to the high heterogeneity of included studies. The overall mean post‐operative Kujala score ranged from 64.3 to 95.3, reported in seven studies comprising 200 patients (261 knees) [1, 18, 20, 22, 40, 41, 50]. Preoperative Kujala scores were reported in three out of seven studies; each of these studies found a significant improvement in Kujala score following surgical management [1, 18, 20]. When stratified by surgical procedure, the mean post‐operative Kujala score from five studies comprising 140 patients (181 knees) who underwent MPFLR ranged from 64.3 to 94 [1, 18, 20, 40, 41], compared to Kujala scores of 81.4 and 95.3, reported in two studies comprising 60 patients (80 knees) who underwent non‐MPFLR procedures [22, 50]. Post‐operative Kujala score following surgical management with autograft utilization was reported in three studies comprising 73 knees. The overall mean post‐operative Kujala scores ranged from 64.3 to 94 [1, 18, 40]. Three studies assessing allografts comprising 109 knees reported post‐operative Kujala scores which ranged from 76.2 to 89 [20, 40, 41].

### Associations with outcomes

Two studies reported the statistical association between Beighton score and patient demographics or post‐operative outcomes [15, 40]. The Beighton score was significantly associated with age, female sex and presence of knee hyperextension, whereas non‐significant statistical associations were reported between the Beighton score and various subjective and objective outcome measures. A higher Beighton score was not correlated with worse post‐operative outcomes or symptom scores. GJH did not correlate with post‐operative functional testing scores and did not impact quality‐of‐life measures [15, 40]. One study reported no significant difference in post‐operative complications between patients with syndromic and non‐syndromic aetiologies of hypermobility [41]. Complete details are found in Table 1.

### Other complications

Across four studies comprising 104 patients, 17 (16.3%) patients were reported to have post‐operative complications at follow‐up, of which 100% are classified as Grade III‐b [5, 20, 22, 40, 41]. The complications included: pain requiring revision surgery (*n* = 7, 41.2%) [40], arthrofibrosis (*n* = 2, 11.8%) [20], marginal skin necrosis (*n* = 2, 11.8%) [22], patellar fracture (*n* = 2, 11.8%) [41], tibial tubercle avulsion and loss of fixation (*n* = 1, 5.9%) [20], surgical site infection (*n* = 1) [20], DVT (*n* = 1, 5.9%) [40] and deformity (*n* = 1, 5.9%) [40].

## DISCUSSION

The primary finding of this review is that surgical management, particularly with MPFLR, of patellar instability in patients with hypermobility results in low redislocation rates. This review demonstrated a weighted mean post‐operative redislocation rate of 9% at the latest follow‐up in patients who underwent any surgical procedure. Patients who underwent MPFLR reported lower rates of redislocation compared to those who underwent any non‐MPFLR surgical procedure.

Redislocation rates vary widely among the most current literature. However, the redislocation rate in hypermobile patients following surgery found in this review ranged from 0% to 22%, which is similar to reported redislocation rates in non‐hypermobile patients, which range from 7% to 25% [3, 6, 9, 27, 30, 37]. Alternatively, redislocation rates in non‐hypermobile patients undergoing conservative treatment range from 35% to 71% [2, 13, 39], indicating that operative treatment in hypermobile patients may be superior to non‐operative treatment in non‐hypermobile patients.

A prior systematic literature review found weak evidence supporting superiority of rehabilitation compared to no‐treatment for hypermobility, and the authors concluded that there is a lack of sufficient literature assessing outcomes of conservative management for hypermobile patients with patellar instability [38]. Systematic reviews report redislocation rates ranging from 30% to 46.4% among non‐hypermobile patients undergoing conservative treatment [3, 6, 9, 27, 30, 37]. While the standard of care for treating patellar instability in hypermobile patients currently favours rehabilitation, it appears that early surgical management, particularly with MPFLR, may result in superior outcomes regarding redislocation rates for this patient population.

One case–control study reported that hypermobile patients who underwent isolated MPFLR experienced satisfactory outcomes, including zero reported redislocation events and significant improvements in Kujala score, Oxford knee score, the Fulkerson modification of Lysholm and Tegner activity scores, and Short‐Form 12 Mental Component Score (SF‐12MCS) [18]. However, functional improvements were significantly inferior compared to patients without hypermobility [18]. In contrast, the results of our systematic review indicate that patients with hypermobility who undergo surgical management for patellar instability experience similar redislocation rates and Kujala scores when compared to non‐hypermobile patients. This may be attributed to the utilized procedure's effectiveness in restoring patellar stability. MPFLR and other patellar realignment procedures (e.g., TTO) address structural risk factors contributing to patellar instability, and their success appears consistent regardless of underlying ligamentous laxity. This suggests that surgical intervention may effectively compensate for the GJH present in these patients.

Moreover, our study found that an increased Beighton score and/or the presence of GJH did not correlate with poorer functional outcomes or quality‐of‐life measures.

While various anatomic risk factors of patellar instability, such as trochlear dysplasia, patella alta and TT‐TG distance, have been thoroughly described in the literature, the pathoanatomy of patellar instability in patients with JHS is not as well understood [15, 16, 20, 25, 41, 42, 48]. Muscle weakness secondary to abnormal collagen function is commonly seen in JHS, and as the MPFL is primarily composed of collagen, these patients may be more susceptible to dislocation. Therefore, MPFLR is a commonly used surgical procedure in this population [14, 16, 25, 43, 53]. Moreover, it is unclear whether utilization of an autograft may result in inferior outcomes in hypermobile patients due to the theoretical hypothesis that their collagen may be defective and weaker in a reconstruction [16]. Among included studies that reported data, 109 knees (39.9%) were managed with surgery utilizing allografts, whereas autografts were used in 164 knees (60.1%). The redislocation rates following surgical management with autografts and allografts were found to be similar. However, data regarding trochlear dysplasia and other anatomic risk factors were not provided. Future research on the relationship between hypermobility and patellar instability is crucial in optimally planning surgical approaches.

The primary limitation of this systematic review was the low quality of the included studies, with the majority of them being level IV evidence. The inclusion of level IV studies which lack comparable treatment arms may introduce bias and heterogeneity [12]. The pooling of results from a range of patient populations, including varying skeletal maturities and chronicity of injuries, introduced significant bias. Furthermore, the majority of studies did not provide preoperative Kujala scores. As such, the direct effects of surgical intervention on Kujala score could not be measured, which may have introduced ‘non‐reporting bias’. Another limitation is due to the inconsistencies in patient demographics across included studies; variations in age, skeletal maturity, preoperative anatomic risk factors, and JHS may have influenced the outcomes. Moreover, results were not stratified by type of JHS, making it difficult to evaluate the optimal surgical technique for each unique patient population. The high heterogeneity of studies assessing redislocation rates following MPFLR reflects the need for future high‐quality comparative studies. Another limitation of this study involves the use of the overall standard deviation (SD) to approximate graft‐specific SDs in one study [40], as these values were not available in the original data; while this approach assumes similar variability between subgroups, potential differences in subgroup variances could impact the precision of the results. The strengths of this review are primarily due to its rigorous methodology; it is a comprehensive analysis of all available studies of surgical management of patellar instability in hypermobile patients. The present study was performed with an extensive systematic search of three medical databases and the use of independent authors to screen and extract data. Furthermore, there was considerable agreement between the authors during the title and abstract stage and during the full‐text stage of screening.

Based on the available data, early MPFLR shows promising outcomes in managing patellar instability in hypermobile patients. We recommend conducting multi‐centre prospective studies with standardized definitions of hypermobility and standardized protocols for surgical procedure, rehabilitation, and follow‐up to more accurately evaluate key outcomes for surgical management of patellar instability in patients with hypermobility.

## CONCLUSION

This systematic review demonstrated that surgical management, particularly MPFLR, of patellar instability in hypermobile patients may result in lower redislocation rates and favourable post‐operative outcomes compared to non‐operative management in non‐hypermobile patients. The current available literature for this patient population is highly heterogeneous, indicating the need for high‐quality studies to more accurately assess intrinsic risk factors and surgical techniques.

## AUTHOR CONTRIBUTIONS

Screening, data extraction, writing and editing: Joshua Dworsky‐Fried and Benjamin Blackman. Writing, editing and idea conception: Dan Cohen and Darren de SA. Writing and editing: Devin Peterson, Olufemi R. Ayeni and Volker Musahl.

## CONFLICT OF INTEREST STATEMENT

The authors declare no conflicts of interest.

## ETHICS STATEMENT

There are no relevant ethical disclosures pertaining to research involving human participants and/or animals, and informed consent was not necessary to develop this manuscript.

## Supporting information

SUPPLEMENTARY DIGITAL MATERIAL hypermobility.docx.

## Data Availability

Data may be made available upon reasonable request at josh.dworsky-fried@medportal.ca.

## References

[1] Abouelsoud MM , Abdelhady A , Elshazly O . Anatomic physeal‐sparing technique for medial patellofemoral ligament reconstruction in skeletally immature patients with ligamentous laxity. Eur J Orthop Surg Traumatol. 2015;25(5):921–926. 25757696 10.1007/s00590-015-1618-1

[2] Bitar AC , Demange MK , D'Elia CO , Camanho GL . Traumatic patellar dislocation: nonoperative treatment compared with MPFL reconstruction using patellar tendon. Am J Sports Med. 2012;40(1):114–122. 22016458 10.1177/0363546511423742

[3] Blackman B , Dworsky‐Fried J , Cohen D , Slawaska‐Eng D , Gyemi L , Simunovic N , et al. Surgical management of first‐time patellar dislocations in paediatric patients may lower rates of redislocation compared to conservative management: a systematic review and meta‐analysis. Knee Surg Sports Traumatol Arthrosc. 2024:1–11. 10.1002/ksa.12524 PMC12104781 39474842

[4] Carter C , Sweetnam R . Familial joint laxity and recurrent dislocation of the patella. J Bone Joint Surg Br. 1958;40‐B(4):664–667. 10.1302/0301-620X.40B4.664 13610980

[5] Clavien PA , Barkun J , De Oliveira ML , Vauthey JN , Dindo D , Schulick RD , et al. The clavien‐dindo classification of surgical complications: five‐year experience. Ann Surg. 2009;250(2):187–196. 19638912 10.1097/SLA.0b013e3181b13ca2

[6] Cohen D , Le N , Zakharia A , Blackman B , De Sa D . MPFL reconstruction results in lower redislocation rates and higher functional outcomes than rehabilitation: a systematic review and meta‐analysis. Knee Surg Sports Traumatol Arthrosc. 2022;30(11):3784–3795. 35616703 10.1007/s00167-022-07003-5

[7] Dietrich T , Fucentese S , Pfirrmann C . Imaging of individual anatomical risk factors for patellar instability. Semin Musculoskelet Radiol. 2016;20(01):65–073. 27077588 10.1055/s-0036-1579675

[8] Duncan ST , Noehren BS , Lattermann C . The role of trochleoplasty in patellofemoral instability. Sports Med Arthrosc. 2012;20(3):171–180. 22878658 10.1097/JSA.0b013e31826a1d37 PMC3964680

[9] Erickson BJ , Mascarenhas R , Sayegh ET , Saltzman B , Verma NN , Bush‐Joseph CA , et al. Does operative treatment of first‐time patellar dislocations lead to increased patellofemoral stability? A systematic review of overlapping meta‐analyses. Arthroscopy. 2015;31(6):1207–1215. 25636989 10.1016/j.arthro.2014.11.040

[10] Fithian DC , Paxton EW , Stone ML , Silva P , Davis DK , Elias DA , et al. Epidemiology and natural history of acute patellar dislocation. Am J Sports Med. 2004;32(5):1114–1121. 15262631 10.1177/0363546503260788

[11] Fulkerson JP , Becker GJ , Meaney JA , Miranda M , Folcik MA . Anteromedial tibial tubercle transfer without bone graft. Am J Sports Med. 1990;18(5):490–497. 2252090 10.1177/036354659001800508

[12] Harris JD , Brand JC , Cote MP , Dhawan A . Research pearls: the significance of statistics and perils of pooling. part 3: pearls and pitfalls of meta‐analyses and systematic reviews. Arthroscopy. 2017;33(8):1594–1602. 28457677 10.1016/j.arthro.2017.01.055

[13] Hawkins RJ , Bell RH , Anisette G . Acute patellar dislocations. Am J Sports Med. 1986;14(2):117–120. 3717480 10.1177/036354658601400204

[14] Heighes LA , Abelleyra Lastoria DA , Beni R , Iftikhar A , Hing CB . The relationship between joint hypermobility and patellar instability: a systematic review. J Orthop. 2024;56:40–49. 38784948 10.1016/j.jor.2024.05.009 PMC11109350

[15] Hiemstra LA , Kerslake S , Kupfer N , Lafave MR . Generalized joint hypermobility does not influence clinical outcomes following isolated MPFL reconstruction for patellofemoral instability. Knee Surg Sports Traumatol Arthrosc. 2019;27(11):3660–3667. 30919002 10.1007/s00167-019-05489-0

[16] Homere A , Bolia IK , Juhan T , Weber AE , Hatch GF . Surgical management of shoulder and knee instability in patients with Ehlers‐Danlos syndrome: joint hypermobility syndrome. Clin Orthop Surg. 2020;12(3):279. 32904109 10.4055/cios20103 PMC7449847

[17] Hopper GP , Heusdens CHW , Dossche L , Mackay GM . Medial patellofemoral ligament repair with suture tape augmentation. Arthrosc Tech. 2019;8(1):e1–e5. 30899643 10.1016/j.eats.2018.08.021 PMC6408510

[18] Howells NR , Eldridge JD . Medial patellofemoral ligament reconstruction for patellar instability in patients with hypermobility: a case control study. J Bone Joint Surg Br. 2012;94‐B(12):1655–1659. 10.1302/0301-620X.94B12.29562 23188907

[19] Hsiao M , Owens BD , Burks R , Sturdivant RX , Cameron KL . Incidence of acute traumatic patellar dislocation among active‐duty united states military service members. Am J Sports Med. 2010;38(10):1997–2004. 20616375 10.1177/0363546510371423

[20] Imerci A , McDonald TC , Rogers KJ , Thacker MM , Atanda A . Outcomes of medial patellofemoral ligament reconstruction and tibial tubercle osteotomy in syndromic adolescents with patellar dislocation. J Clin Orthop Trauma. 2022;25:101770. 35127438 10.1016/j.jcot.2022.101770 PMC8803613

[21] Insall J , Falvo K , Wise D . Chondromalacia patellae. A prospective study. J Bone Joint Surg. 1976;58(1):1–8. 1249094

[22] Joo SY , Park KB , Kim BR , Park HW , Kim HW . The ‘four‐in‐one’ procedure for habitual dislocation of the patella in children: early results in patients with severe generalised ligamentous laxity and aplasis of the trochlear groove. J Bone Joint Surg Br. 2007;89‐B(12):1645–1649. 10.1302/0301-620X.89B12.19398 18057367

[23] Khormaee S , Kramer DE , Yen Y‐M , Heyworth BE . Evaluation and management of patellar instability in pediatric and adolescent athletes. Sports Health. 2015;7(2):115–123. 25984256 10.1177/1941738114543073 PMC4332641

[24] Kujala UM , Jaakkola LH , Koskinen SK , Taimela S , Hurme M , Nelimarkka O . Scoring of patellofemoral disorders. Arthroscopy. 1993;9(2):159–163. 8461073 10.1016/s0749-8063(05)80366-4

[25] Kutschke MJ , Albright JA , Winschel JM , He EW , Cruz AI , Daniels AH , et al. Increased risk of patellofemoral instability events and surgical management in patients with joint hypermobility syndromes: a matched cohort analysis. Arthrosc Sports Med Rehabil. 2024;6(6):100995. 39776511 10.1016/j.asmr.2024.100995 PMC11701986

[26] Landis JR , Koch GG . The measurement of observer agreement for categorical data. Biometrics. 1977;33(1):159. 843571

[27] Le N , Blackman B , Zakharia A , Cohen D , De Sa D . MPFL repair after acute first‐time patellar dislocation results in lower redislocation rates and less knee pain compared to rehabilitation: a systematic review and meta‐analysis. Knee Surg Sports Traumatol Arthrosc. 2023;31(7):2772–2783. 36372845 10.1007/s00167-022-07222-w

[28] Lee D‐Y , Kang D‐G , Jo H‐S , Heo S‐J , Bae J‐H , Hwang S‐C . A systematic review and meta‐analysis comparing conservative and surgical treatments for acute patellar dislocation in children and adolescents. Knee Surg Relat Res. 2023;35(1):18. 37349852 10.1186/s43019-023-00189-z PMC10286373

[29] Liberati A , Altman DG , Tetzlaff J , Mulrow C , Gøtzsche PC , Ioannidis JPA , et al. The PRISMA statement for reporting systematic reviews and meta‐analyses of studies that evaluate health care interventions: explanation and elaboration. Ann Intern Med. 2009;151(4):W‐65–W‐94. 10.7326/0003-4819-151-4-200908180-00136 19622512

[30] Longo UG , Ciuffreda M , Locher J , Berton A , Salvatore G , Denaro V . Treatment of primary acute patellar dislocation: systematic review and quantitative synthesis of the literature. Clin J Sport Med. 2017;27(6):511–523. 28107220 10.1097/JSM.0000000000000410

[31] Malek S , Reinhold EJ , Pearce GS . The Beighton Score as a measure of generalised joint hypermobility. Rheumatol Int. 2021;41(10):1707–1716. 33738549 10.1007/s00296-021-04832-4 PMC8390395

[32] Merchant AC , Mercer RL . Lateral release of the patella: a preliminary report. Clin Orthop Relat Res. 1974;103:40–45. 10.1097/00003086-197409000-00027 4414065

[33] Minghao L , Zhenxing S , Qiang L , Guoqing C . Lateral retinacular release for treatment of excessive lateral pressure syndrome: the Capsule‐Uncut Immaculate (cui) technique. Arthrosc Tech. 2023;12(11):e1991–e1996. 38094964 10.1016/j.eats.2023.07.018 PMC10714330

[34] Monllau JC , Erquicia JI , Ibañez M , Gelber PE , Ibañez F , Masferrer‐Pino A , et al. Reconstruction of the medial patellofemoral ligament. Arthrosc Tech. 2017;6(5):e1471–e1476. 29354460 10.1016/j.eats.2017.06.039 PMC5710065

[35] Niedzielski KR , Malecki K , Flont P , Fabis J . The results of an extensive soft‐tissue procedure in the treatment of obligatory patellar dislocation in children with ligamentous laxity: a post‐operative isokinetic study. Bone Joint J. 2015;97‐B(1):129–133. 10.1302/0301-620X.97B1.33941 25568426

[36] Nomura E , Inoue M , Kobayashi S . Generalized joint laxity and contralateral patellar hypermobility in unilateral recurrent patellar dislocators. Arthroscopy. 2006;22(8):861–865. 16904584 10.1016/j.arthro.2006.04.090

[37] Nwachukwu BU , So C , Schairer WW , Green DW , Dodwell ER . Surgical versus conservative management of acute patellar dislocation in children and adolescents: a systematic review. Knee Surg Sports Traumatol Arthrosc. 2016;24(3):760–767. 26704809 10.1007/s00167-015-3948-2

[38] Palmer S , Davey I , Oliver L , Preece A , Sowerby L , House S . The effectiveness of conservative interventions for the management of syndromic hypermobility: a systematic literature review. Clin Rheumatol. 2021;40(3):1113–1129. 32681365 10.1007/s10067-020-05284-0 PMC7895781

[39] Palmu S , Kallio PE , Donell ST , Helenius I , Nietosvaara Y . Acute patellar dislocation in children and adolescents: a randomized clinical trial. J Bone Joint Surg Am. 2008;90(3):463–470. 18310694 10.2106/JBJS.G.00072

[40] Parikh SN , Nemunaitis J , Wall EJ , Cabatu C , Gupta R , Veerkamp MW . Midterm outcomes of isolated medial patellofemoral ligament reconstruction for patellar instability in Ehlers‐Danlos syndrome. Orthop J Sports Med. 2024;12(6):23259671241241096. 38845609 10.1177/23259671241241096 PMC11155334

[41] Reddy G , Hayer PS , UlIslam S , Mehta NJ , Iqbal HJ , Stables G , et al. Outcomes of allograft medial patellofemoral ligament reconstruction in children and adolescents with hypermobility. Int J Appl Basic Med Res. 2022;12(3):161–166. 36131861 10.4103/ijabmr.ijabmr_25_22 PMC9484516

[42] Redler LH , Dennis ER , Mayer GM , Kalbian IL , Nguyen JT , Shubin Stein BE , et al. Does ligamentous laxity protect against chondral and osteochondral injuries in patients with patellofemoral instability? Orthop J Sports Med. 2022;10(7):23259671221107609. 35833196 10.1177/23259671221107609 PMC9272185

[43] Rombaut L , Malfait F , De Wandele I , Taes Y , Thijs Y , De Paepe A , et al. Muscle mass, muscle strength, functional performance, and physical impairment in women with the hypermobility type of Ehlers‐Danlos syndrome. Arthr Care Res. 2012;64(10):1584–1592. 10.1002/acr.21726 22556148

[44] Schöttle PB , Fucentese SF , Pfirrmann C , Bereiter H , Romero J . Trochleaplasty for patellar instability due to trochlear dysplasia: a minimum 2‐year clinical and radiological follow‐up of 19 knees. Acta Orthop. 2005;76(5):693–698. 16263617 10.1080/17453670510041781

[45] Shelbourne K , Urch S , Gray T . Results of medial retinacular imbrication in patients with unilateral patellar dislocation. J Knee Surg. 2012;25(5):391–396. 23150348 10.1055/s-0032-1313750

[46] Slim K , Nini E , Forestier D , Kwiatkowski F , Panis Y , Chipponi J . Methodological index for non‐randomized studies (*MINORS*): development and validation of a new instrument. ANZ J Surg. 2003;73(9):712–716. 12956787 10.1046/j.1445-2197.2003.02748.x

[47] Smits‐Engelsman B , Klerks M , Kirby A . Beighton score: a valid measure for generalized hypermobility in children. J Pediatr. 2011;158(1):119–123.e4. 20850761 10.1016/j.jpeds.2010.07.021

[48] Thompson P , Metcalfe AJ . Current concepts in the surgical management of patellar instability. Knee. 2019;26(6):1171–1181. 31787447 10.1016/j.knee.2019.11.007

[49] Tobias JH , Deere K , Palmer S , Clark EM , Clinch J . Joint hypermobility is a risk factor for musculoskeletal pain during adolescence: findings of a prospective cohort study. Arthr Rheum. 2013;65(4):1107–1115. 23450628 10.1002/art.37836

[50] Trisolino G , Depaoli A , Gallone G , Ramella M , Olivotto E , Zarantonello P , et al. A 20‐year retrospective study of children and adolescents treated by the three‐in‐one procedure for patellar realignment. J Clin Med. 2023;12(2):702. 36675630 10.3390/jcm12020702 PMC9861102

[51] Vasiliadis AV , Troupis T , Chrysikos D , Chytas D , Noussios G . Anatomic risk factors for patellofemoral joint instability: an infographic as a visual learning tool. Cureus. 2024;16(1):e53170. 38420044 10.7759/cureus.53170 PMC10901470

[52] Vellios EE , Trivellas M , Arshi A , Beck JJ . Recurrent patellofemoral instability in the pediatric patient: management and pitfalls. Curr Rev Musculoskelet Med. 2020;13(1):58–68. 31983043 10.1007/s12178-020-09607-1 PMC7083998

[53] Veteto A , McIntyre M , Hintz M , Cramberg M , Kondrashov P . Histological structure of the medial and lateral patellofemoral ligaments and implications for reconstructive surgery and anterior knee pain. Mo Med. 2023;120(2):134–138. 37091936 PMC10121125

[54] West SL , Gartleehner G , Mansfield AJ . Comparative effectiveness review methods: clinical heterogeneity [Internet]. Rockville, MD: Agency for Healthcare Research and Quality (US); 2010. Table 7, Summary of common statistical approaches to test for heterogeneity. 21433337

[55] Wolf JM , Cameron KL , Owens BD . Impact of joint laxity and hypermobility on the musculoskeletal system. J Am Acad Orthop Surg. 2011;19:463–471. 21807914 10.5435/00124635-201108000-00002

[56] Zhao J , Huangfu X , He Y , Liu W . Recurrent patellar dislocation in adolescents: medial retinaculum plication versus vastus medialis plasty. Am J Sports Med. 2012;40(1):123–132. 21900625 10.1177/0363546511420551

