# Supplementary material for: Outcomes following surgical management of patellar instability in hypermobile patients are favourable compared to non‐operative management in non‐hypermobile patients: A systematic review and meta‐analysis
Source: J Exp Orthop. 2025 Jun 1;12(2):e70256. doi: 10.1002/jeo2.70256 (PMC12126757; doi:10.1002/jeo2.70256)
Supplement: Supplementary file 1 — SUPPLEMENTARY DIGITAL MATERIAL hypermobility.docx. [file JEO2-12-e70256-s001.docx]

**SUPPLEMENTARY DIGITAL MATERIAL**

**Supplementary Table 1. Search Criteria**

| **Search Criteria** |
| --- |
| 1. MPFL OR medial patellofemoral ligament |
| 1. Patellar instability OR patellar dislocation |
| 1. Repair OR reconstruction OR treatment |
| 1. Connective tissue disorder OR hyperflexibility OR joint instability OR hypermobile OR hypermobility OR laxity OR Ehlers-Danlos syndrome |
| 1. #1 AND #2 AND #3 AND #4 |
